# A block randomised controlled trial of a brief intervention with a cohort of first year hospitality trainees

**DOI:** 10.1186/1940-0640-8-S1-A58

**Published:** 2013-09-04

**Authors:** Ann M Roche, Ken Pidd, Jane Fischer

**Affiliations:** 1 National Centre for Education and Training on Addiction (NCETA), Flinders University, Adelaide South Australia, Australia

## 

Targeted interventions for high risk groups are increasingly important. Alcohol problems are not evenly distributed. Highly differentiated patterns and prevalence have been identified among workers according to their industry and occupational groups. Young workers in the hospitality industry have been found to be among the highest consumers of alcohol and drugs yet largely overlooked for early and brief intervention. To assess the efficacy of an innovative brief intervention on risky drinking and associated psycho-social measures a trial was undertaken with a cohort of Australian first year hospitality trainees. The study comprised a block randomised controlled trial. Baseline data (T1) was collected at the commencement of hospitality training from both control and intervention groups prior to the administration of a brief intervention and 5 months later at the end of the first term of training (T2). The intervention focused on strategies to address workplace social norms and pressures, resilience and coping strategies, alternative stress management techniques, assertiveness training and social support mechanisms. The intervention was informed by a systematic review of the literature and a qualitative study involving focus groups. Measures included AUDIT, K10, quality of life measures and life satisfaction scales. The findings indicate high levels of risky alcohol consumption among this group of young workers. Significant relationships between age, gender and psycho-social measures were found for risky drinkers. The results of brief intervention indicated changes in the desired direction. Findings are presented together details of efficacy, acceptability to students, implications for wide scale implementation and the feasibility of sustained interventions of this type. This is one of few brief intervention RCTs of its type that address a high risk population using a tailored and targeted intervention and offers considerable promise.

